# Synthesis of enantiomerically enriched (*R*)-^13^C-labelled 2-aminoisobutyric acid (Aib) by conformational memory in the alkylation of a derivative of L-alanine

**DOI:** 10.3762/bjoc.7.152

**Published:** 2011-09-20

**Authors:** Stephen P Fletcher, Jordi Solà, Dean Holt, Robert A Brown, Jonathan Clayden

**Affiliations:** 1 School of Chemistry, University of Manchester, Oxford Rd., Manchester M13 9PL, UK

**Keywords:** amino acid, asymmetric synthesis, conformational memory, isotopic label, isotopomer, quaternary stereogenic centre

## Abstract

The method of Kouklovsky and coworkers for the enantioselective alkylation of cyclic *N*-naphthoyl derivatives of amino acids was used to introduce a ^13^C label into one of the two enantiotopic methyl groups of 2-aminoisobutyric acid (Aib) by retentive alkylation of L-alanine with ^13^CH_3_I. Conditions were identified for optimization of yield and enantiomeric purity, and the absolute configuration of the labelled product was established.

## Introduction

**Figure 1:**
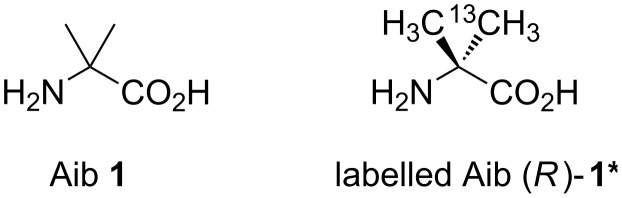
2-Aminoisobutyric acid (Aib).

In connection with our work on the control of conformation in helical foldamers [1–3] built from quaternary α-amino acids [4–5], we required a reliable source of 2-aminoisobutyric acid (Aib) **1** (Figure 1) bearing an isotopically labelled methyl group in enantiomerically enriched form, i.e., (*R*)- or (*S*)-1* or its derivatives. Specifically for our purposes, a high degree of enantiomeric enrichment was not necessary, since we were aiming to make mixtures containing detectable quantities of unequal pairs of diastereoisomeric derivatives. We have previously made isotopically labelled anilines [6] for related studies on oligo-urea foldamers [7–9]. Achiral quaternary amino acids such as Aib and its substituted congeners are powerful promoters of 3_10_ helix formation in peptides [10–13]. But since these helices lack the intrinsic bias towards a single screw-sense, which characterizes typical peptide helices, they are ideal candidates for studying the potential of conformational control in extended systems. Aib is furthermore a common component of several fungal and bacterial antibiotic metabolites [14], and the helix-forming properties of these metabolites, which encourage them to embed themselves into cell membranes, are at the origin of their biological activity.

The methods available for the asymmetric synthesis of quaternary amino acids [15–20] fall principally into three classes. In the first, exemplified by the method of Schöllkopf [21–24], a diastereoselective alkylation is used to relay the stereochemistry of an existing stereogenic centre to the new quaternary centre. In the second, exemplified by the asymmetric phase transfer reactions of Maruoka [25], the alkylation takes place under the influence of a catalyst or other intermolecular influence. In the third strategy, an amino acid is alkylated without recourse to an external controlling influence. Instead the stereochemistry of the amino acid is first used to control a temporary stereogenic element (a centre or axis), which does not appear in the final product. Alkylation through a planar enolate then takes place under the control of the temporary axis or centre in a sequence where the final structure retains a “chiral memory” (in fact a “configurational memory”) of the starting material. The strategy was first developed, in the guise of the “self-regeneration of stereocentres” by Seebach [26–29], but has more recently been shown by Fuji and Kawabata [30–34], by Kouklovsky and coworkers [35–36] and by others [21,37–39] to work effectively when the temporary stereogenic element [40] is a C–N or C–C axis, which retains its configuration at low temperature but becomes conformationally unrestricted as the temperature rises [41–45].

Our interest in the control and exploitation of conformation, particularly in amides and related functional groups [4–5,46–51], and its conceptual link with the wider aims of our work, led us to explore the potential of this last strategy. In particular, we made use of the method of Kouklovsky [36], which relies on a temporary naphthamide Ar–CO axis as a chiral “aide-mémoire” for translating the configuration of the starting amino acid into the configuration of the product. We have previously employed benzamide and naphthamide Ar–CO axes in “chiral memory” processes [52–53]. Kouklovsky reported the enantioselective alkylation of alanine by this method [36], and recently Soai showed that Seebach’s methods can be used to make selectively deuterated Aib [54]. In a previous paper [55], we reported the synthesis and use of a ^13^CH_3_-labelled Aib probe made by Kouklovsky’s method [36], to gather evidence of helicity switching in Aib oligomers. We now report in detail a practical method for the enantioselective synthesis and enantiomeric assignment of a derivative of (*R*)-1*.

## Results and Discussion

**Scheme 1:**
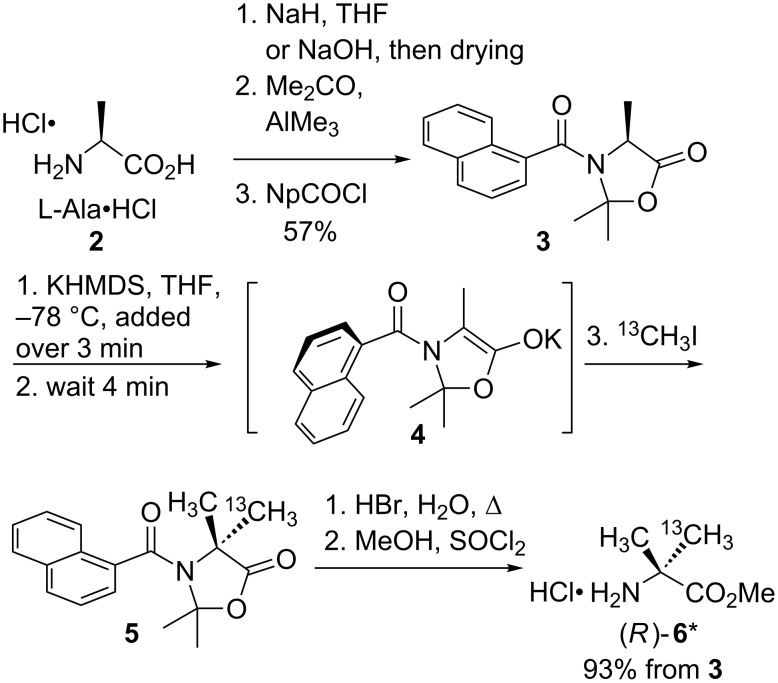
Alkylation of L-alanine.

L-Alanine hydrochloride **2** was converted to its sodium salt either in situ with two equiv sodium hydride, or (preferably, and more conveniently for storage in quantity) by treating with sodium hydroxide and drying for several days under vacuum. The latter method avoided the presence of yellow impurities in the final amino acid. The alaninate salt was converted to its cyclic naphthamide derivative **3**, following the method of Branca et al. [36], resulting in 57% yield and >99.5:0.5 e.r. on a 2.5 g scale (Scheme 1). As ^13^C labelled MeOTf was unavailable, we decided to alkylate **3** with ^13^CH_3_I, despite the poorer results reported [36] with halide leaving groups. Optimal conditions are shown in Scheme 1: The best enantiomeric ratios in the final products (see below) were obtained by adding 2 equiv of a precooled THF solution of KHMDS (potassium hexamethyldisilazide) over 3 min to a THF solution of the naphthamide **3**, allowing a further 4 min for complete deprotonation, and then quenching at –78 °C with an excess of ^13^CH_3_I. By this method, (*R*)-6* was obtained in 93% yield on a 400 mg scale, as a 2.1:1 ratio of enantiomers.

The products of related alkylations are reported [36] to arise from the diastereoselective alkylation of the conformationally locked enolate **4** whose stereochemistry results from the selective deprotonation of the less hindered conformer of **3**. Alkylated **5** was freed from its protecting group and the naphthamide “aide-mémoire” by heating to reflux in 47% hydrobromic acid, followed by esterification with methanol and thionyl chloride, which returned the labelled Aib as its methyl ester hydrochloride salt 6* in 93% yield from **3**. The ^1^H spectrum of 6* in CD_3_OD consisted of a 3H CO_2_Me singlet at 3.85 ppm, plus two coincident signals centred on 1.47 ppm: A 3H doublet (^3^*J*_HC_ = 4.3 Hz) for the unlabelled Me, and a 3H doublet (^1^*J*_HC_ = 130 Hz) for the labelled Me.

**Scheme 2:**
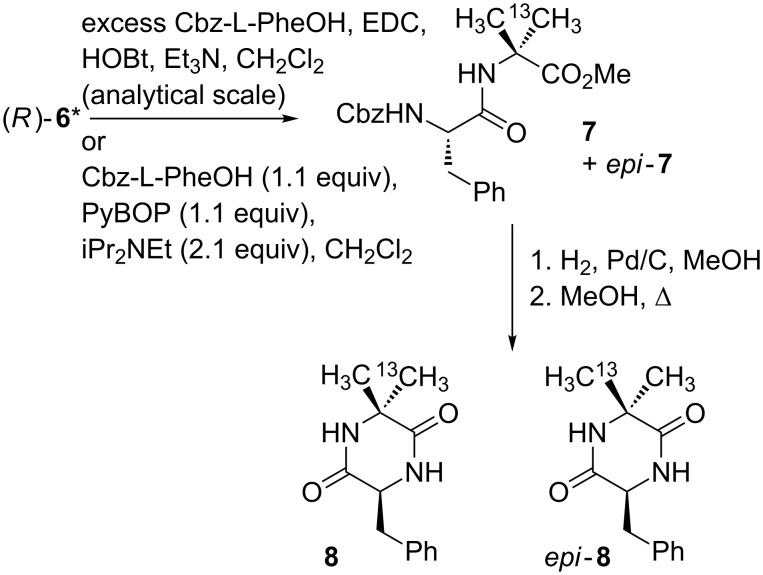
Determining enantiomeric purity and absolute configuration of 6*.

**Figure 2:**
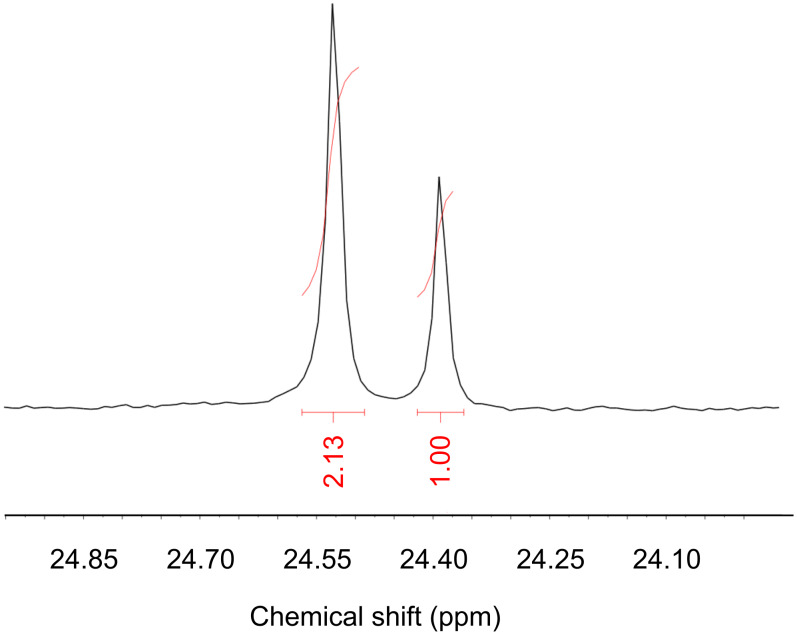
Portion of ^13^C NMR spectrum of **7** showing 2.1:1 ratio of diastereoisomeric isotopomers.

The enantiomeric purity of the products was quantified by formation of pairs of diastereoisomeric dipeptides from small aliquots of 6* with an excess of Cbz-L-Phe, EDC and HOBt (Scheme 2). The ratio of diastereoisomers was determined by comparison of the peak heights of the two ^13^C signals by ^13^C NMR (Figure 2).

As shown in Table 1, the d.r. of **7** (and hence the e.r. of the products **5** and 6*) was strongly dependent on the conditions of the reaction and in particular on the time allowed between deprotonation of **3** and the addition of ^13^CH_3_I. Presumably, shorter reaction times minimise the extent of racemization of the intermediate enolate **4** [36]. Addition of the base over a period of three minutes was essential to avoid warming of the sample, and the delay before adding the electrophile could be reduced to 4 min without a decrease in the yield. Isolated yields of **6** increased to >90% on a larger scale.

**Table 1:** Dependence of yield and e.r. on delay before alkylation.

Time *t* (Scheme 2)/min	Isolated yield **6**/%	d.r. of **7**

8	70	1.2:1
8	80	1.4:1
5	80	1.8:1
4	78	2.1:1

**Figure 3:**
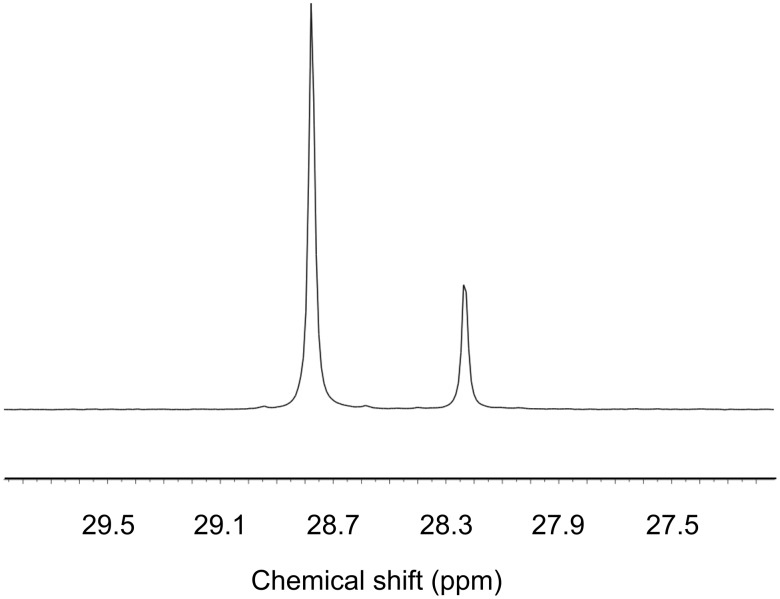
Portion of ^13^C NMR spectrum of **8** showing location of ^13^C label in the less shielded methyl group.

**Figure 4:**
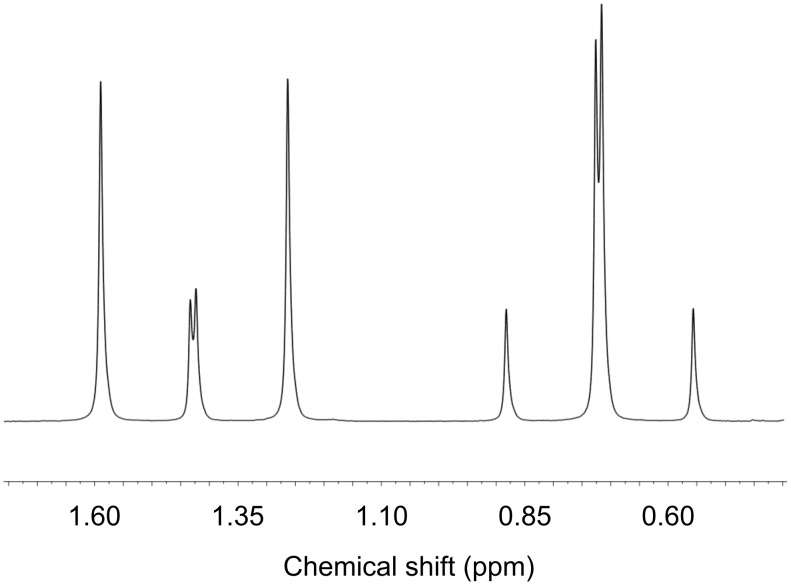
Portion of ^1^H NMR spectrum of **8** showing greater ^1^*J*_HC_ coupling in the less shielded methyl group.

Using the method of Woodard [56], we were able to confirm that the alkylation of (*S*)-Ala by this method is mechanistically retentive, producing (*R*)-6* as the major enantiomer. Dipeptide **7** was made, as a ca. 2.5:1 mixture of diastereoisomeric isotopomers, on a preparative scale from enantiomerically enriched 6*. The dipeptide was deprotected by hydrogenation and cyclized by refluxing in methanol [56] to yield diketopiperazine **8**. The major isotopomer displayed a ^13^C label in the more downfield of the two diastereotopic methyl groups (Figure 3) indicating that the more shielded methyl group, *cis* to the phenyl ring, is the principally unlabelled one. Likewise, in the ^1^H NMR spectrum, the less shielded methyl signal exhibits a ^1^*J*_HC_ coupling of greater magnitude than the ^3^*J*_HC_ coupling, indicating a greater proportion of ^13^C in the less shielded methyl group, *trans* to the phenyl ring (Figure 4).

By this method we were able to obtain ^13^C-labelled, protected Aib (*R*)-6* for use in the synthesis, on gram scale, of spectroscopic probes for helicity [56].

## Experimental

**(*S*)-2,2-Dimethyl-4-methyl-3-(1-naphthoyl)-oxazolidin-5-one (3)**: By a modification of the method of Branca [36,57], sodium hydride (575 mg of a 60% dispersion in mineral oil, 12.4 mmol) was suspended in 5 mL of dry THF. L-Alanine hydrochloride (1.28 g, 10.2 mmol) in 5 mL of THF was slowly added and the mixture was stirred for 2 h. The THF was removed under reduced pressure to yield the sodium salt.

Alternatively, L-alanine (8.9 g, 0.1 mol) was dissolved in 1 M NaOH (100 mL), evaporated to dryness and dried for several days in a vacuum oven. The sodium alaninate formed in this way was highly hygroscopic, and a portion (1.6 g, 14.4 mmol) was transferred to the reaction flask under a stream of argon.

To the sodium alaninate salt, 40 mL of dry acetone and 11.0 g of powdered molecular sieves were added. The mixture was cooled to 0 °C, and trimethylaluminium (7.20 mL of a 2.0 M solution in toluene, 14.4 mmol) was added dropwise under vigorous stirring. The mixture was allowed to reach room temperature and stirred overnight. The reaction mixture was cooled to 0 °C and 1-naphthoyl chloride (2.16 mL, 14.4 mmol) was added by syringe. The mixture was stirred for a further 2.5 h, filtered through a pad of Celite^®^, washed with diethyl ether and concentrated in vacuo. The crude product was purified by column chromatography (SiO_2_, EtOAc/Petrol 20:80) to yield 2.30 g (8.11 mmol, 57%) of the desired compound as white solid. The enantiomeric ratio was >99:1 (Chiralpak AD-H, hexane/ethanol: 95/5, 1.0 mL/min: *t*_R_ (*S*) = 28.4 min, *t*_R_ (*R*) = 31.1 min. Spectroscopic data was consistent with that reported in the literature, apart from the CHN proton, which appears to be erroneously reported [36].

*R*_f_: 0.40 (SiO_2_, EtOAc/Petrol 20:80); ^1^H NMR (500 MHz, CDCl_3_) δ 0.99 (s, 3H), 2.05 (s, 3H), 2.10 (s, 3H), 4.16 (brs, 1H), 7.50 (m, 4H), 7.84 (brm, 1H), 7.90–7.96 (m, 2H) ppm; ^13^C NMR (125 MHz, CDCl_3_) δ 20.0 (CH_3_), 26.1 (CH_3_), 27.5 (CH_3_), 53.8 (CH), 98.4 (C), 124.2 (CH), 124.5 (CH), 124.9 (CH), 126.7 (CH), 127.8 (CH), 128.8 (CH), 130.4 (CH), 133.50 (C), 133.54 (C), 167.6 (CO), 171.4 (CO) ppm.

(*R*)-^13^C-Aminoisobutyric acid methyl ester hydrochloride salt [(*R*)-6*]: Following the method reported in the supporting information of reference [55], potassium hexamethyl disilazide (11.7 mL, 0.5 M in toluene 5.88 mmol) in a flame dried pear-shaped flask was evaporated to dryness. THF (20 mL) was added through a septum, and the suspension was agitated for several minutes, then cooled to –78 °C. The cold mixture was then quickly added (syringe, 5 × 4 mL portions, over 3 min) to a stirred solution of **3** (793 mg, 2.80 mmol) at –78 °C in THF (20 mL). After 4 min ^13^CH_3_I (0.87 mL, 13.99 mmol) was added in one portion. The color changed from deep red to pale yellow. After a further 30 min, HCl (1 N, 10 mL) was added. The reaction mixture was allowed to warm to room temperature, partitioned between water and ethyl acetate, and the organic phase was concentrated. HBr (47% in water, 20 mL) was added and the mixture was heated to reflux for 18 h, then cooled to room temperature, diluted with water and washed twice with CH_2_Cl_2_ and once with ether. The aqueous phase was evaporated to dryness. Methanol (20 mL) and thionyl chloride (2 mL) were added and the reaction mixture heated to reflux for 1 h. The reaction mixture was concentrated, and excess SOCl_2_ was removed by adding toluene and concentrating to dryness, giving 6* (400 mg, 93%) as waxy crystals, mp 168–170 °C (dec.) [lit. for unlabelled **6** 182–183 °C (dec.) [58]; lit. for doubly labelled 6** 180–182 °C (dec.) [55]].

^1^H NMR (CD_3_OD, 400 MHz) δ 1.57 (d, ^4^*J*_CH_ = 4.3 Hz 3H), 1.57 (d, ^1^*J*_CH_ = 130 Hz, 3 H), 3.85 (s, 3H); ^13^C NMR (CD_3_OH, 100 MHz) δ 24.2 (two superimposed singlets), 54.8 (d, *J* = 9.0 Hz), 58.0 (d, *J* = 35.4 Hz), 174.1; IR ν_max_: 3365, 2919, 1743, 1506, 1318, 1175 cm^–1^; MS (ES^+,^ MeOH): 141 ([M + Na]^+^, 60%), 119 ([M + H]^+^, 100%). HRMS (ES^+^, MeOH): Calcd for C_4_^13^C_1_H_11_N_1_O_2_ + H: 119.0902; found, 119.0865.

Coupling of 6* with Cbz-L-Phe to yield 7 (analytical scale): Following the method described in the supporting information of reference [55], an excess of Cbz-L-phenylalanine (40 mg, 0.13 mmol), HOBt (20 mg, 0.2 mmol), EDC (0.03 mL, 0.2 mmol) and Et_3_N (0.3 mL) were added to a suspension of 6* (4 mg, 0.03 mmol) in CH_2_Cl_2_ (4 mL). The resulting solution was stirred overnight at room temperature and partitioned between water and ethyl acetate. The organic layer was washed with water, NaHCO_3_ × 2, 1 N HCl × 2, and again with water, dried over MgSO_4_, and concentrated. All remaining organic material was dissolved in CDCl_3_ and the d.r. of **7** was determined by integration of the methyl signals (24.52 ppm and 24.36 ppm) in the ^13^C NMR spectrum.

Coupling of 6* with Cbz-L-Phe to yield 7 (preparative scale): 6* (50 mg, 0.33 mmol) was suspended in DCM (5 mL), and to this was added Cbz-L-phenylalanine (106 mg, 0.35 mmol) and PyBOP (186 mg, 0.35 mmol). The mixture was cooled to 0 °C. DIPEA (0.12 mL, 0.68 mmol) was added dropwise and the resulting solution stirred overnight at room temperature. NaHCO_3_ (sat. 5 mL) was added, followed by DCM (5 mL) and the layers were separated. The aqueous layer was extracted with DCM, and the combined organic extracts washed with brine, dried over MgSO_4_ and concentrated in vacuo. The crude product was purified by column chromatography (SiO_2_, EtOAc/Petrol 20:80) to yield 88 mg (0.22 mmol, 68 %) of the desired compound as a white solid.

*R*_f_: 0.23 (SiO_2_, EtOAc/Petrol 20:80); ^1^H NMR (400 MHz, CDCl_3_) δ 1.41 (d, *J* = 4.3 Hz, 3H^minor^), 1.41 (d, *J* = 130 Hz, 3H^major^), 1.44 (d, *J* = 4.3 Hz, 3H^major^), 1.44 (d, *J* = 130 Hz, 3H^minor^), 2.99 (dd, *J* = 7.5, 13.7 Hz, 1H), 3.15 (dd, *J* = 5.6, 13.5 Hz, 1H), 3.71 (s, 3H), 4.37 (bm, 1H), 5.11 (bs, 2H), 5.38 (d, *J* = 6.6 Hz, 1H), 6.13 (s, 1H), 7.21–7.39 (m, 10H); ^13^C NMR (100 MHz, CDCl_3_) δ 24.4 (minor), 24.5 (major), 52.7, 56.2 (d, *J* = 1.85 Hz), 56.3, 56.7, 67.0, 127.1, 128.0, 128.2, 128.5, 128.7, 129.5, 169.7, 174.5; IR ν_max_: 3278, 3064, 2948, 1742, 1703, 1658, 1547 cm^–1^; MS (ES^+^, MeOH): 422 ([M + Na]^+^, 100%), 400 ([M + H]^+^, 20%). HRMS (ES^+^, MeOH): Calcd for C_21_^13^C_1_H_27_N_2_O_5_ + Na^+^: 400.1949; found, 400.1960.

**Deprotection and cyclization of 7 to yield diketopiperazine 8: 7** (59 mg, 0.15 mmol) was dissolved in MeOH (2 mL) under a nitrogen atmosphere. 10% Pd/C (6 mg) was carefully added and the mixture stirred under an atmosphere of hydrogen until complete consumption of starting material, as determined by TLC (24 h). The mixture was washed through a pad of Celite^®^ with EtOAc and concentrated in vacuo. The residue was subsequently redissolved in MeOH (2 mL) and refluxed for 48 h. On cooling to room temperature a white precipitate appeared which was isolated by filtration to yield 10 mg (0.043 mmol 30%) of the desired product as a white solid.

^1^H NMR (400 MHz, F_3_CCO_2_D) δ 0.70 (d, *J* = 4.0 Hz, 3H^major^), 0.70 (d, *J* = 132 Hz, 3H^minor^), 1.41 (d, *J* = 4.0 Hz, 3H^minor^), 1.41 (d, *J* = 132 Hz, 3H^major^), 3.10 (dd, *J* = 4.5, 14.4 Hz, 1H), 3.25 (dd, *J* = 4.8, 14.4 Hz, 1H), 4.67 (t, *J* = 4.6 Hz, 1H), 7.07 (d, *J* = 4.0 Hz, 2H), 7.15–7.26 (m, 3H); ^13^C NMR (100 MHz, CDCl_3_) δ 28.2 (minor), 28.8 (major), 41.4, 58.6, 130.4, 131.2, 132.0, 135.0, 172.1, 176.6; IR ν_max_: 3180, 3035, 2994, 2974, 2960, 2931, 2897, 1660, 1605, 1453 cm^–1^; MS (ES^+,^ MeOH): 256 ([M + Na]^+^, 100%); HRMS (ES^+^, MeOH): Calcd for C_12_^13^C_1_H_16_N_2_O_2_ + Na^+^: 234.1319; found, 234.1311.
